# Arboviral diseases and poverty in Alabama, 2007–2017

**DOI:** 10.1371/journal.pntd.0009535

**Published:** 2021-07-06

**Authors:** Donal Bisanzio, Elisa Martello, Katherine Izenour, Kelly Stevens, Ramandeep Kaur, Benjamin A. McKenzie, Moritz Kraemer, Richard Reithinger, Sarah Zohdy

**Affiliations:** 1 RTI International, Washington, District of Columbia, United States of America; 2 Division of Epidemiology and Public Health, School of Medicine, University of Nottingham, Nottingham, United Kingdom; 3 Auburn University College of Veterinary Medicine, Auburn, Alabama, United States of America; 4 Alabama Department of Public Health, Montgomery, Alabama, United States of America; 5 Auburn University School of Forestry and Wildlife Services, Auburn, Alabama, United States of America; 6 Department of Pediatrics, Harvard Medical School, Boston, Massachusetts, United States of America; 7 Computational Epidemiology Lab, Boston Children’s Hospital, Boston, Massachusetts, United States of America; 8 Department of Zoology, University of Oxford, Oxford, United Kingdom; Faculty of Science, Ain Shams University (ASU), EGYPT

## Abstract

Mosquito-borne viruses cause diseases of great public health concern. Arboviral disease case distributions have complex relationships with socioeconomic and environmental factors. We combined information about socio-economic (population, and poverty rate) and environmental (precipitation, and land use) characteristics with reported human cases of arboviral disease in the counties of Alabama, USA, from 2007–2017. We used county level data on West Nile virus (WNV), dengue virus (DENV), chikungunya virus (CHIKV), Zika virus (ZIKV), California serogroup virus, Eastern equine encephalitis virus, and Saint Louis encephalitis virus to provide a detailed description of their spatio-temporal pattern. We found a significant spatial convergence between incidence of WNV and poverty rate clustered in the southern part of Alabama. DENV, CHIKV and ZIKV cases showed a different spatial pattern, being mostly located in the northern part, in areas of high socioeconomic status. The results of our study establish that poverty-driven inequities in arboviral risk exist in the southern USA, and should be taken into account when planning prevention and intervention strategies.

## Author summary

Mosquito-borne arboviruses like West Nile virus (WNV), dengue virus (DENV), chikungunya virus (CHIKV), Zika virus (ZIKV), California serogroup virus (CSV), Eastern equine encephalitis virus (EEE), and Saint Louis encephalitis virus (SLE) are on the rise globally. Socioeconomic and environmental conditions have played a role in directing in this expansion by creating conditions ideal for mosquito vectors and transmission. In this study, we used 10 years (2007–2017) of county level human arboviral case data from the US state of Alabama to better understand the roles socioeconomics (poverty rate) and environmental (land use, precipitation, land cover) conditions may play in driving patterns of arboviral disease in the southern US. We found a significant association between poverty rate and incidence of WNV, an arbovirus primarily transmitted by *Culex* spp. mosquitoes, which are known for thriving in contaminated water sources and sewage overflow. Conversely, cases of DENV, CHIKV, and ZIKV, arboviruses primarily transmitted by *Aedes* spp. mosquitoes, were reported in areas of high socioeconomic status. These findings suggest differential distribution of arboviruses relevant to human health in Alabama, and that poverty in the southern US is a significant factor that should be considered when planning WNV prevention and intervention strategies.

## Introduction

Globally, infectious diseases are recognized as leading causes of mortality for the world’s poor [1]. The association between infectious diseases and socio-economic status, specifically poverty, is well established, and it has been shown to be strongest when examining vector-borne and parasitic diseases [2]. Mosquito-borne diseases have complex, non-linear relationships with factors such as climate, socio-economics, hydrology, policy, and access to health care [3]. For example, poor civil infrastructure leads to more standing water and tire dumps, which are ideal mosquito breeding habitats [4]. Similarly, cases of chikungunya virus (CHIKV) recorded at the beginning of the outbreak in French Guiana in 2016 were associated with low-income neighborhoods [5].

Studies investigating the association between socio-economic factors and mosquito-borne infectious diseases in the USA have reported contrasting results. While middle income neighborhoods show higher transmission risk of West Nile virus (WNV) in the northern USA [6,7], in the South, neighborhoods of low socio-economic status show higher transmission risk [8–11]. To our knowledge, no study has described the association between socio-economic factors and mosquito-borne diseases in Alabama. Due to the state’s heterogeneous distribution of socio-economics and physiographic characteristics, Alabama provides ideal conditions to investigate such association. Circulation of arboviruses, such as WNV and eastern equine encephalitis virus (EEEV), sustained by local population of mosquitoes has been recorded in Alabama [12,13]. In recent years, *Aedes aegypti*, a main vector of several arboviral diseases, including CHIKV, Zika (ZIKV), and dengue (DENV) fever, has been detected in Alabama after a 26-year absence [14]. The state’s physiographic regions and ecological conditions are ideal habitats for *Aedes aegypti* and *Ae*. *albopictus* [15,16], which thrive when breeding sites expand following extreme weather events such as tropical storms and hurricanes. Finally, over ten million residents in the Gulf States—including Alabama—live below the USA poverty line [17], making it the USA’s most impoverished region [18,19].

Here, we use publicly available data from 2007–2017 to examine socio-ecological associations between human incidence of mosquito-borne arboviral disease, county level indicators of poverty, and mosquito vector distributions. Our aim was to evaluate whether an association between poverty level and mosquito-borne diseases occurs in the southeastern USA, specifically Alabama.

## Material & methods

### Data dictionary

#### Human case data

This paper analyzes human cases of seven arboviral diseases reported in Alabama’s counties from 2007–2017 (data provided by the Alabama Department of Public Health): California serogroup virus (CSV), Eastern equine encephalitis virus (EEEV), Saint Louis encephalitis virus (SLEV), West Nile virus (WNV), DENV, ZIKV, and CHIKV. We used cases officially reported by each Alabama county during that time period. Cases are reported by county of patient’s residence and not necessarily by county of disease acquisition. Detailed case definition is described in the S1 Appendix.

#### Demographic and environmental data

Data on poverty, and population at the county level were obtained from the USA Census Bureau (https://www.census.gov). We used the all-age poverty rate from the year 2007 to 2017 calculated by the Small Area Income and Poverty Estimates (SAIPE) Program (https://www.census.gov/programs-surveys/saipe.html). The SAPEI Program applies estimates the number of people living below the poverty threshold at the county level for all the USA using regression models based on census data. The poverty threshold is set by the Census Bureau based on family size and age of the members (https://www.census.gov/topics/income-poverty/poverty/guidance/poverty-measures.html). Population data included population of all ages from the year 2007 to the year 2017. To account for environmental factors, we downloaded land use data at county level from the USA Department of Agriculture (https://www.ers.usda.gov/data-products/major-land-uses/). From these data, we extracted the fraction of each county’s territory covered by woodland, crops, water bodies, and urban areas. Data on rainfall was provided by Emory University, consisting of a multi-band raster on the monthly mean of daily accumulated rainfall from 2007–2017 at 30 km of resolution. We collected data on flooding events from the Federal Emergency Management Agency’s (FEMA) through the OpenFEMA platform (https://www.fema.gov/media-library/collections/). We included flooding events in our analyses because—if occurring—they could increase the mosquito breeding sites and contribute to an increase in the risk of arboviral infection for humans [20]. A map of *Ae*. *aegypti* predicted presence for Alabama was extracted from the raster map obtained from the Malaria Atlas Project’s data repository (https://malariaatlas.org/) [16] (Fig D in S1 Appendix).

#### Statistical analysis

We performed statistical analyses to investigate the relationship of poverty rate with human WNV, DENV, ZIKV, and CHIKV cases. Given the high number of WNV cases reported from 2007–2017, we calculated and used annual incidence in our statistics (Table 1). EEEV, SLEV, and CSV cases were not included in the analyses due to the low number of reported cases (Table 1 and Fig A in S1 Appendix). We assessed the association between WNV incidence and poverty rate (POOR) through a generalized linear model (GLM) with Gaussian likelihood adjusted for environmental characteristics (ENV), precipitation (RAIN), flooding events (FLOODING), and spatial autocorrelation (SPAT). The regression model was performed for each year from 2007 to 2017. The environmental characteristics included in the model were: percentage of territory occupied by urban areas, crops, woodlands, and water bodies. Model formula:

WNVIncidence=POOR+ENV+RAIN+FLOODING+SPAT

**Table 1:** Reported Arboviruses disease cases by disease agent from 2007 to 2017 in Alabama. California serogroup virus (CSV), Eastern equine encephalitis virus (EEEV), Saint Louis encephalitis virus (SLEV), West Nile virus (WNV), Dengue virus (DENV), Chikungunya virus (CHIKV), Zika virus (ZIKV) human cases were divided in two groups by competent mosquito vector genus (*Culex* spp. and *Aedes* spp.).

Arbovirus	2007	2008	2009	2010	2011	2012	2013	2014	2015	2016	2017	Tot
*Zoonotic arboviruses*												
CSV	-	-	1	-	1	-	2	-	-	-	-	4
EEEV	1	1	-	-	-	-	-	1	-	-	-	3
SLEV	-	-	-	-	1	-	-	1	-	-	1	3
WNV	24	18	-	3	5	62	9	2	9	18	61	211
*Non-zoonotic arboviruses*												
DENV	5	2	1	4	4	4	5	3	3	5	-	36
CHIKV	-	-	-	-	-	-	-	19	1	1	1	22
ZIKV	-	-	-	-	-	-	-	-	-	41	4	45

- no human cases were reported

We also performed a GLM to investigate the relationship of poverty rate (POOR), predicted presence of *Ae*. *aegypti* and aggregated DENV, ZIKV and CHIKV cases across years, from 2007–2017. We aggregated cases belonging to the three viruses because these were all declared as imported cases. The model was adjusted by population (POP), predicted presence of *Ae*. *aegypti* (VECTOR), and spatial autocorrelation (SPAT). Model formula:

DENV/ZIKV/CHIKVcases=VECTOR+POOR+SPAT

Given the high number of non-reporting counties (68.7%) and the heterogeneity of DENV, ZIKV, and CHIKV case counts, we have modeled these cases using a zero-inflated negative binomial as the selected distribution. Environmental factors were not included in the model because we assumed that the *Ae*. *aegypti* presence map already accounted for them.

The spatial autocorrelation was accounted in both the GLMs by using the spatial Gaussian Markov random field (GMRF) models [21]. Spatial statistic was applied to analyze the spatial pattern of WNV incidence. The Getis’ Gi*(d) local statistic was used to identify hot-spots of WNV incidence in Alabama [22]. The spatial weight used to perform the spatial statistic test was based on the inverse of distance among all counties. Significance was evaluated by comparing observed values with values expected under the null hypothesis of complete spatial randomness (based on 999 Monte Carlo permutations of county status). A power analysis test was conducted to assess the statistical power of each regression model with a medium effect size (d = .50) and with alpha of .05. Only regression model showing a statistical power > 0.8 were performed [23]. Significance was set as p<0.05 and p<0.01. Collinearity among model variables was investigated by calculating the variance inflation factors (VIF) [24]. A VIF = 1 indicates no correlation among the variables. A VIF between 1 and 5 suggests that there is a moderate correlation, but it is not severe enough to affect the model. A model showing a VIF>5 indicates highly correlated variables, and it needs some correction. Maps and statistical analysis were performed using the R software [25]. The geodata was managed using QGIS [26].

## Results

**Fig 1:**
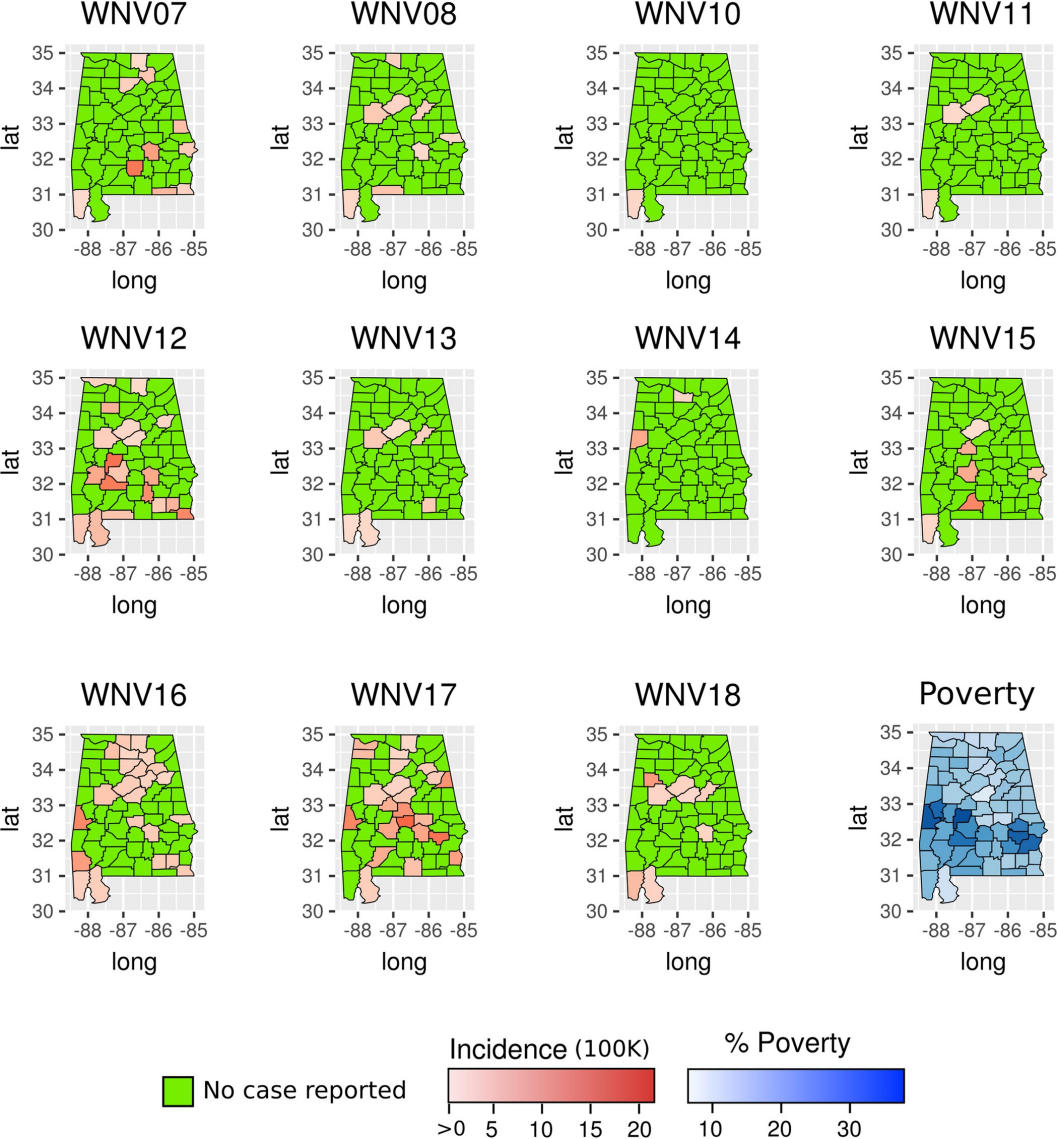
Incidence of West Nile Virus (WNV) at county level in Alabama from 2007 to 2017. The maps show the years when human cases were reported. The figure also includes the percentage of the population living in low socio-economic status at county level based on census data. The maps were created using public available county boundaries downloaded from www.usgs.gov processed with the R software [25].

A total of 324 arboviral disease cases were reported in Alabama from 2007–2017 (Table 1; detailed summary of cases divided by county and year is available in S1 Appendix). Among the reported cases, 65.1% (211 cases) were due to WNV infection. ZIKV, DENV and CHIKV accounted for 13.9% (45), 11.1% (36) and 6.8% (22) of cases, respectively. The remaining 3.1% of cases were due to SLEV (3 cases), EEEV (3 cases), and CSV (4 cases). Cases of SLEV, EEEV, and CSV were not included in the analyses due to the low number and no evident spatial pattern (Table 1 and Fig A in S1 Appendix). High circulation of WNV was recorded during 2012 and 2017 when the incidence was high in Alabama’s southern counties (Fig 1).

**Fig 2:**
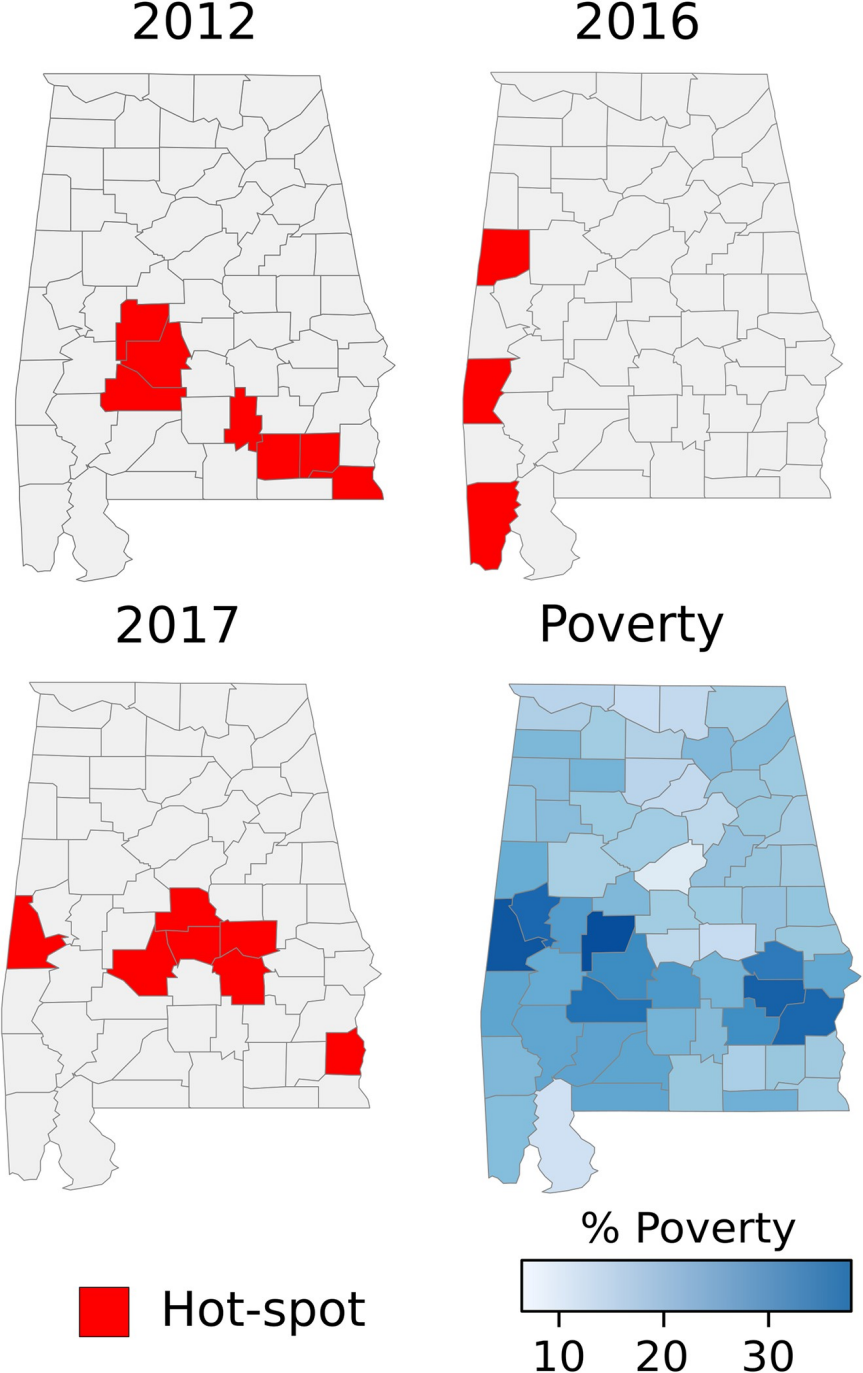
Hot-spots of WNV incidence at county level in Alabama in the years 2012 and 2017. The maps show the three years when outbreaks of WNV occurred. The figure also includes the percentage of the population living in low socio-economic status at the county level based on census data. The maps were created using public available county boundaries downloaded from www.usgs.gov processed with the R software [25].

**Fig 3:**
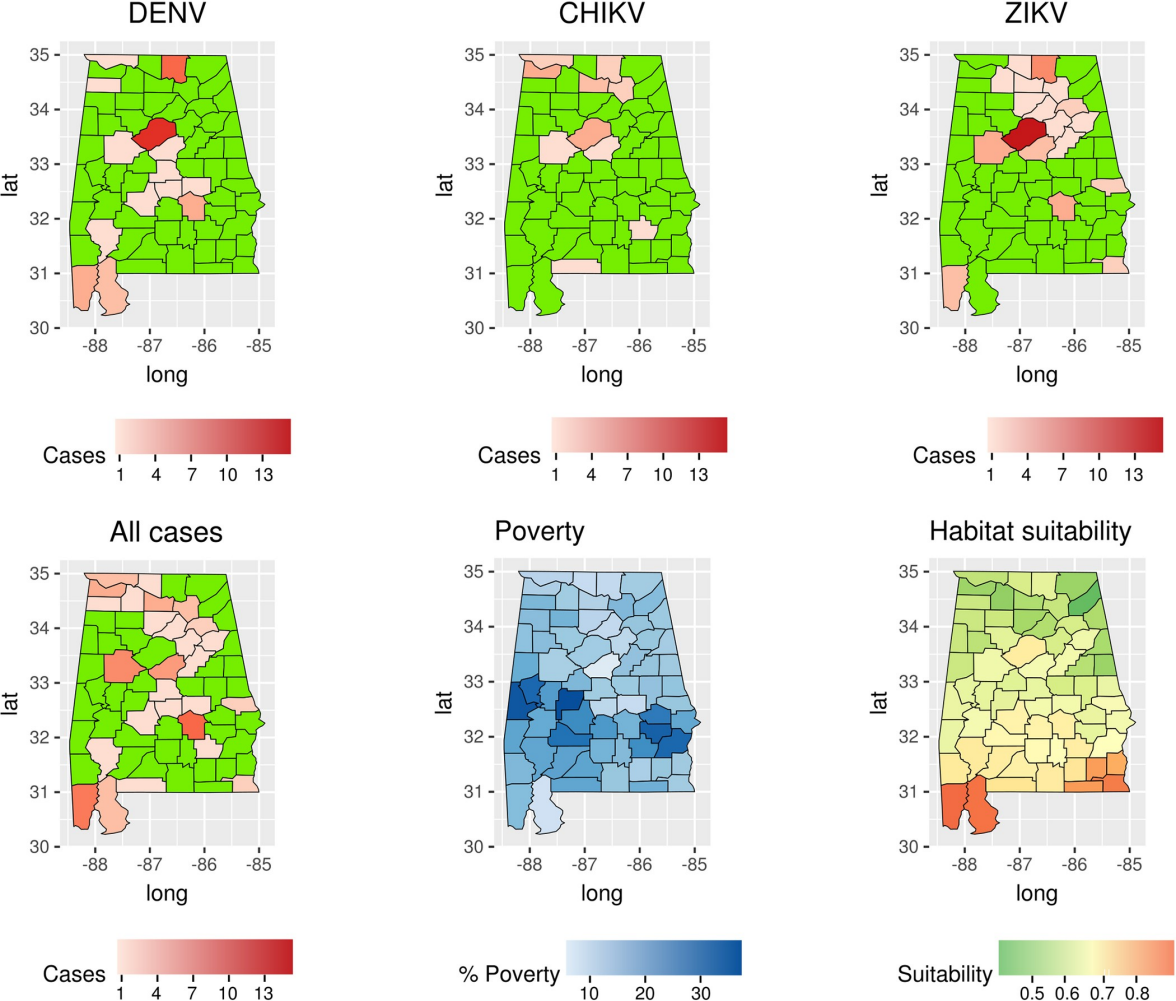
Number of DENV, CHIKV, ZIKV, and total cases at county level in Alabama from 2007 to 2017. The map shows the years when cases were reported. The figure includes the percentage of the population living in low socio-economic status and vector habitat suitability at county level based on census data. The maps were created using public available county boundaries downloaded from www.usgs.gov processed with the R software [25].

Indeed, the Gi*(d) identified several hot-spots in those counties (Fig 2). In contrast, during the 2009, 2011, 2013, 2015 and 2016, few WNV cases were reported in the state (Table 1). In addition, our regression model showed that WNV incidence was positively correlated with the fraction of population living under the poverty threshold, after controlling for population, flooding events, precipitation, and land use (Table 2). Specifically, this association was positive during the high circulation years (p<0.05), but negative during 2008 (p<0.05) (Table 2). Poverty was also positively associated with WNV incidence during 2007, although not significantly. DENV cases were recorded during every year prior to 2017 (Table 1 and Fig B in S1 Appendix). Most of the cases were reported from counties in northern Alabama. A similar spatial pattern was found for ZIKV and CHIKV cases (Fig 3 and Fig C in S1 Appendix). CHIKV and ZIKV cases showed a spike during their first reporting year (2014 for CHIKV and 2016 for ZIKV), accounting for more than 90% of all reported cases (Table 1 and Fig C in S1 Appendix). The number of cases for these three diseases combined was negatively correlated with poverty (GLM, p<0.05, Table 3). Predicted presence of *Ae*. *aegypti* was not associated to the number of reported cases of DENV, ZIKV, and CHIK (GLM, p>0.05, Table 3 and Fig 3). None of the regression models showed a VIF>5 indicating a small correlation among the variables.

**Table 2:** Yearly association between West Nile Virus (WNV) incidence and socio-economic status at county level in Alabama. The association was estimated using regression model adjusted by land use, rainfall, flooding events, and population density. No WNV cases were reported during the year 2009 in Alabama.

Year	Socio-economic status	VIF
2007	Positive association	2.3
2008	Negative association**	2.6
2010	-	
2011	-	
2012	Positive association*	2.8
2013	-	
2014	-	
2015	-	
2016	Positive association**	2.7
2017	Positive association**	2.3

- model power <0.8

*p<0.05

**p<0.01

**Table 3:** Association of DENV, CHIKV, ZIKV cases with socio-economic status, and *Ae aegypti* habitat suitability. The table shows the result of the GLM. The power of the model was >0.8 and the model VIF was equal to 3.2.

Covariates	DENV/CHIKV/ZIKV association
Socio-economic status	Negative association*
*Ae*. *aegypti* predicted presence	No association

*p<0.05

## Discussion

Using spatio-temporal analytics, we provide a comprehensive picture of human arboviral disease burden and distribution in Alabama from 2007–2017. Additionally, we explored the association of these cases with socio-economic status at county level. The highest number of arboviral cases reported in Alabama was due to WNV, with two outbreaks occurring in 2012 and 2017.

The influence of socioeconomically-linked human behaviors and physical neighborhood characteristics on the presence of arboviral vectors and infection rates is complex and varied, interacting with relatively fine-scale environmental characteristics. Recent research suggests that decaying infrastructure and neighborhood abandonment moderate the influence of local vegetation and precipitation on distributions of WNV vector mosquitoes among some urban mosquito populations, but only during times of normal to high levels of precipitation [27]. However, only mean neighborhood income, not infrastructure or abandonment, were associated with WNV infection in these same urban mosquito populations [28]. Arboviral infection, specifically incidence of SLEV, DENV and western equine encephalitis (WEE), in the US has also traditional been tied to socioeconomic indicators such as use of air-conditioning and television utilization, with greater use of these indoor services providing a protective effect against arboviral infection [29,30]. The more indoor lifestyle associated with the use of air-conditioning and television limits exposure to arboviral vectors. Such socioeconomically-linked behavioral modifications are harder to spatially quantify than physical neighborhood characteristics, which may explain why socioeconomic status itself may be a better spatial predictor of arboviral infection than some environmental factors.

During WNV outbreak years in Alabama, incidence hot-spots were identified in the southern part of the state, an area where poverty rate is the highest. Indeed, our results showed significant spatial convergence between WNV incidence and poverty within Alabama. This finding is in alignment with previous studies performed in Illinois, Michigan, and California, which have suggested that socio-economic status is the greatest predictor of WNV transmission and occurrence of cases at the county level [7,8]. The association between WNV and poverty may be due to the greater availability of mosquito breeding habitats and housing quality in low-income neighborhoods. Low-income areas are more likely to contain vacant lots, antiquated sewage infrastructure, and other breeding habitats for mosquitoes [3,8].

Living in low-income areas generally increases exposure to infectious diseases, including non-vector-borne diseases. A recent study found gastrointestinal parasites present in more than the 30% of at-risk population in Lowndes County, Alabama, despite several interventions having been carried out in the county [31]. Lowndes is located in a low-income region of the central part of Alabama characterized by low taxes on property, high rates of poverty and unemployment, low-achieving schools, and high rates of out-migration [32]. Our study reiterates the need of a prioritizing public health intervention in low-income areas, given that the population living in these areas is at greater risk of several diseases, including arboviral infections such as WNV.

Compared to other arboviruses, the low prevalence of EEEV, SLEV, and CSV in Alabama is in line with national trends over the past several years [33]. The discrepancy in transmission rates between WNV and other viruses may be explained by competitive exclusion between antigenically similar arboviruses that share host species, an exclusion which may have led to a decrease in transmission of viruses like SLEV in areas where WNV has become endemic [34]. However, in recent years SLEV has appeared to have re-emerged in the Americas [35] and there is evidence of co-circulation of SLEV and WNV. In 2015, concurrent outbreaks of WNV and SLEV were detected in the southwestern US in Arizona [36], suggesting potential for ongoing co-circulation of these arboviruses in the US. While human clinical cases of EEEV, SLEV, and CSV are uncommon, they retain great importance to public health due either to high case fatality rates (EEEV) or history of causing epidemics in the USA (SLEV) [35,37]. The finding could also be due to different transmission dynamics of the viruses compared to WNV, the very different habitats in which the virus transmission could occur and different preferred hosts and primary vectors [38]. For example, EEEV is endemic in bottomland hardwood swamps and *Culiseta melanura*, the main mosquito vector, feeds almost exclusively on birds with a low probability of causing human spillover [38]. In addition, the transmission dynamics and the low human infection rate of EEV, SLEV, and CSV reported here could be justified by several factors characterizing the county—these include the availability and abundance of competent and incompetent reservoir hosts, vector feeding preferences and behavior, and the interactions between these species [39].

The occurrence of DENV, CHIKV, and ZIKV cases in Alabama showed a spatial pattern different from WNV cases. Indeed, these three viruses were mostly located in northern Alabama, i.e. generally high-income areas and main transportation hubs. Several studies have explored the negative association of these diseases with socio-economic status, albeit with differing conclusions [40]. For example, cases of CHIKV and ZIKV recorded at the beginning of an outbreak in French Guiana, where *Ae*. *aegypti* are common and where environmental conditions support local transmission, were associated with low-income neighborhoods [5]. In Alabama, where environmental conditions are not as favorable for local transmission of CHIKV and ZIKV, cases were imported from endemic areas due to travelling [41]. This can be one of the factors explaining the negative association of poverty rate with occurrence of DENV, CHIKV, and ZIKV cases. Low-income population has lower international mobility compared to fraction of the population due to limited economic resources. High numbers of cases of CHIKV, during 2014, and ZIKV, during 2015, were recorded concurrently with transmission outbreaks in Central and South America [42–44]. Although most DENV, CHIKV, and ZIKV cases were reported in counties with low probability of *Ae*. *aegypti* presence, cases were also recorded in the coastal counties with high habitat suitability for the vector. Consequently, autochthonous transmission could establish itself there, an event that has already happened repeatedly in Florida [45]. The high habitat suitability for the vector estimated in southern Alabama increases the risk of possible establishment of viruses spread by *Ae*. *aegypti* in areas with high levels of poverty.

The main limitation of our analyses is that cases reported by the Alabama Department of Public Health (ADPH) rely on passive surveillance and reporting from local physicians and county health departments. Actual incidence of disease is likely under-reported, with detection and reporting of neuroinvasive diseases being considered more consistent and complete than that of non-neuroinvasive diseases. Diagnostic testing approaches and the potential lack of a differential diagnosis may be another factor impacting reported case rates. Another limitation of our study is that we did not include entomological data in the WVN regression models. The study relied on the environmental characteristics of the counties to capture the variability of the abundance of *Culex* spp. mosquitoes across Alabama and transmission risk. One of the limitations affecting our study is the difficulties of low-income population in accessing health services. This could have resulted in under-reporting of arboviral disease cases. The comparison among WNV disease, a zoonotic disease, and dengue, chikungunja, and Zika, human-specific diseases, could be limited by the different ecological and immunological processes causing cases in human. Despite these limitations, the strong association between poverty and WNV hotspots shown in our study emphasizes the significance of identifying and adjusting for socio-economic risk factors when developing vector control and arboviral disease intervention plans.

The high numbers of WNV cases in Alabama and their clear association with poverty are indicative of a need for a paradigm shift in the control of arboviral disease, specifically WNV, in the southern USA. The Gulf states are home to over 15 million people living below the poverty line, more than anywhere else in the nation. However, with regards to arboviral disease control, many of these states do not receive the adequate resources proportionate to the disease risk and burden. In high income countries, populations living in poverty, like those in the US Gulf states, are highly vulnerable to arboviral infection, as well as to other neglected tropical diseases (NTDs)[17]: however, these populations do not receive scientific or public health support proportional to the burden of disease they are experiencing. Evidence of bi-directional associations between socio-economic status and vector-borne diseases are well-established in low and lower-middle income countries. The results of our study establish that poverty-driven arboviral inequities are present in the southern USA, and should be taken into account as a part of the public health decision, budgeting and response making process.

## Supporting information

S1 Appendix The appendix includes: alabama case definition for arboviral diseases; incidence maps of Easter Equine Encephalitis virus (EEEV), California serogroup virus (CSV), Saint Louis encephalitis virus (SLEV), dengue cases (DENV), zika (ZIKV), and chicungunya (CHIKV) at county at county level in Alabama from 2007 to 2017; map showing presence probability of *Ae*. *aegypti* in 2015. The map was created using available county boundaries downloaded from www.usgs.gov and data from the paper written by Kraemer and colleagues [16] (available at: https://malariaatlas.org/) processed with the R software [25].
(DOCX)
