# Supplementary material for: Arboviral diseases and poverty in Alabama, 2007–2017
Source: PLoS Negl Trop Dis. 2021 Jul 6;15(7):e0009535. doi: 10.1371/journal.pntd.0009535 (PMC8284636; doi:10.1371/journal.pntd.0009535)
Supplement: S1 Appendix — The map was created using available county boundaries downloaded from www.usgs.gov and data from the paper written by Kraemer and colleagues [16] (available at: https://malariaatlas.org/) processed with the R software [25]. (DOCX) [file pntd.0009535.s001.docx]

**Technical appendix**

**Alabama case definition for arboviral diseases**

In Alabama, arboviral disease cases are categorized into two groups: neuroinvasive disease and non-neuroinvasive disease. There were no restrictions on what cases were modeled in this study. For neuroinvasive disease, a “case” is one that meets clinical criteria for neuroinvasive disease and has supportive lab evidence. For nonneuroinvasive disease a “case” is one that meets clinical criteria for nonneuroinvasive disease and has supportive lab evidence. The case definitions for Zika virus disease, Zika virus infection without disease, non-congenital infections were those that meet clinical criteria for non-congenital disease and have laboratory evidence of ZIKV infection by: culture, viral antigen or viral RNA in serum, CSF, tissue, or other specimen or a positive ZIKV IgM antibody test of serum or CSF with positive ZIKV neutralizing antibody titers and negative neutralizing antibody titers against dengue or other flaviviruses endemic to the region where exposure occurred.

**Figures**


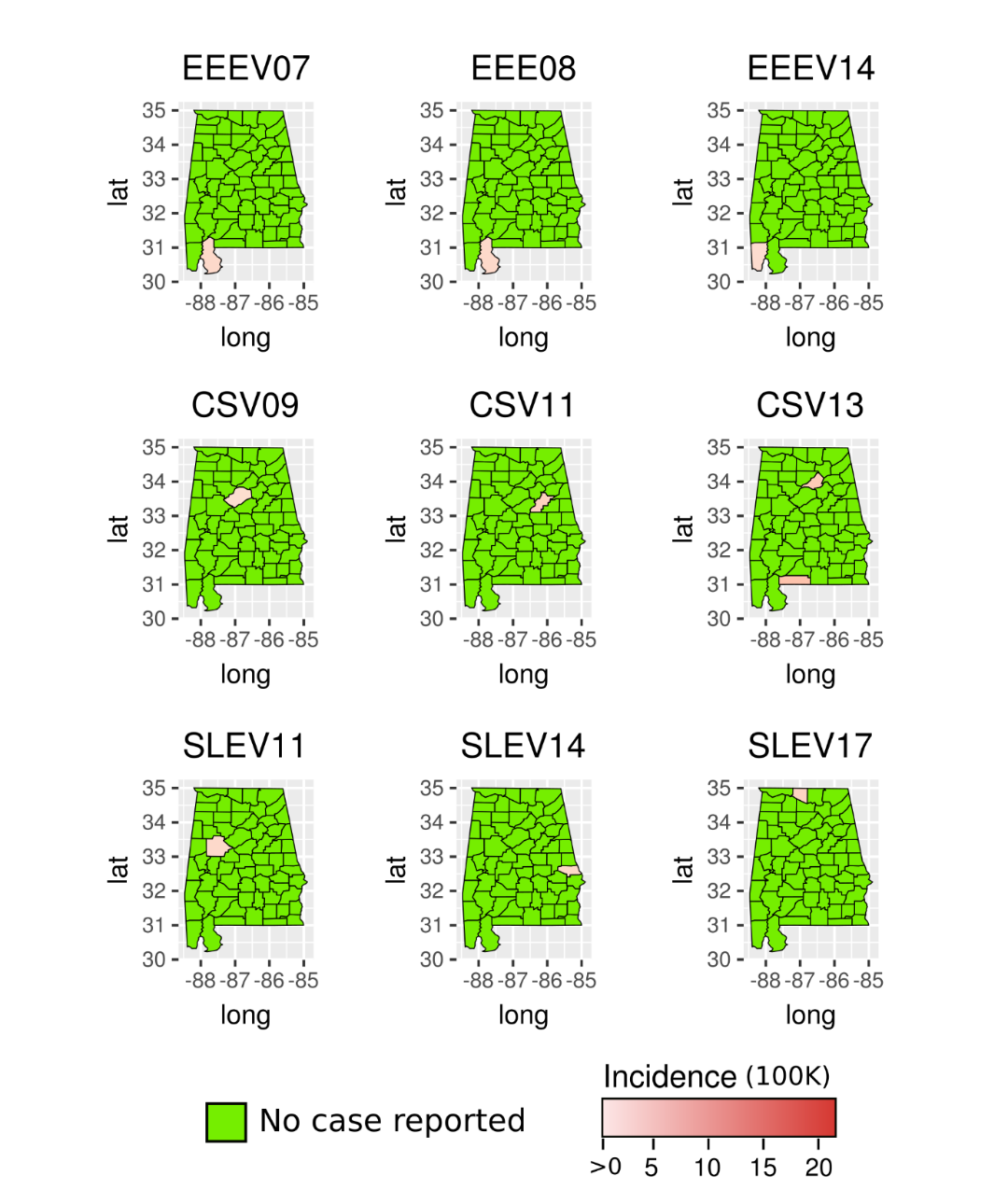


**Fig A. Incidence of Easter Equine Encephalitis virus (EEEV), California serogroup virus (CSV), and Saint Louis encephalitis virus (SLEV) at county level in Alabama from 2007 to 2017.** The maps show incidence of EEEV, CSV and SLEV in the years when cases were reported. The maps were created using public available county boundaries downloaded from [www.usgs.gov](http://www.usgs.gov) processed with the R software [1].

**
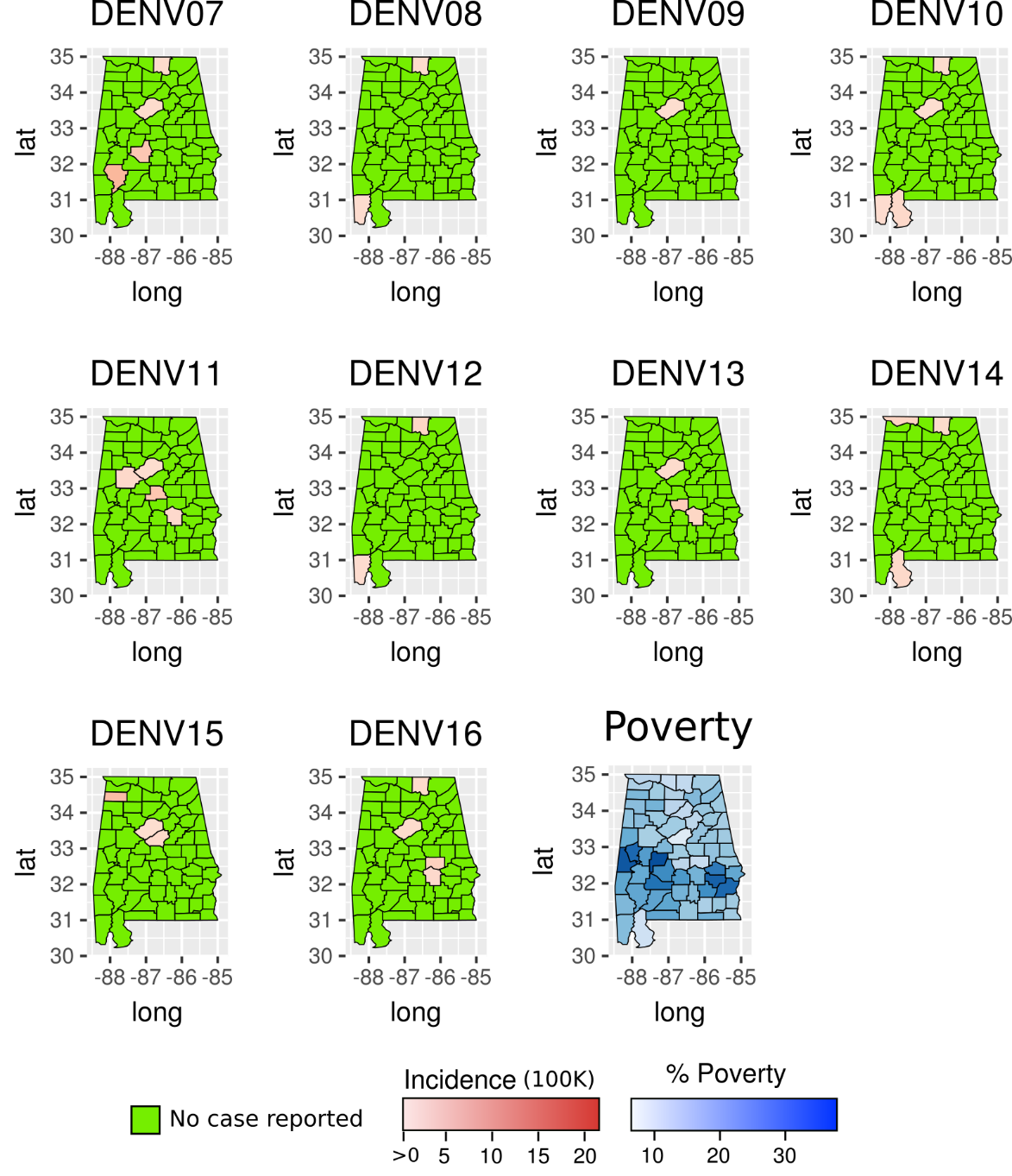
**

**Fig B. Incidence of dengue cases (DENV) at county level in Alabama from 2007 to 2017.** The maps show incidence of DENV in the years when cases were reported. The figure also includes the percentage of the population living in low socio-economic status at county level based on census data. The maps were created using public available county boundaries downloaded from [www.usgs.gov](http://www.usgs.gov) processed with the R software [1].


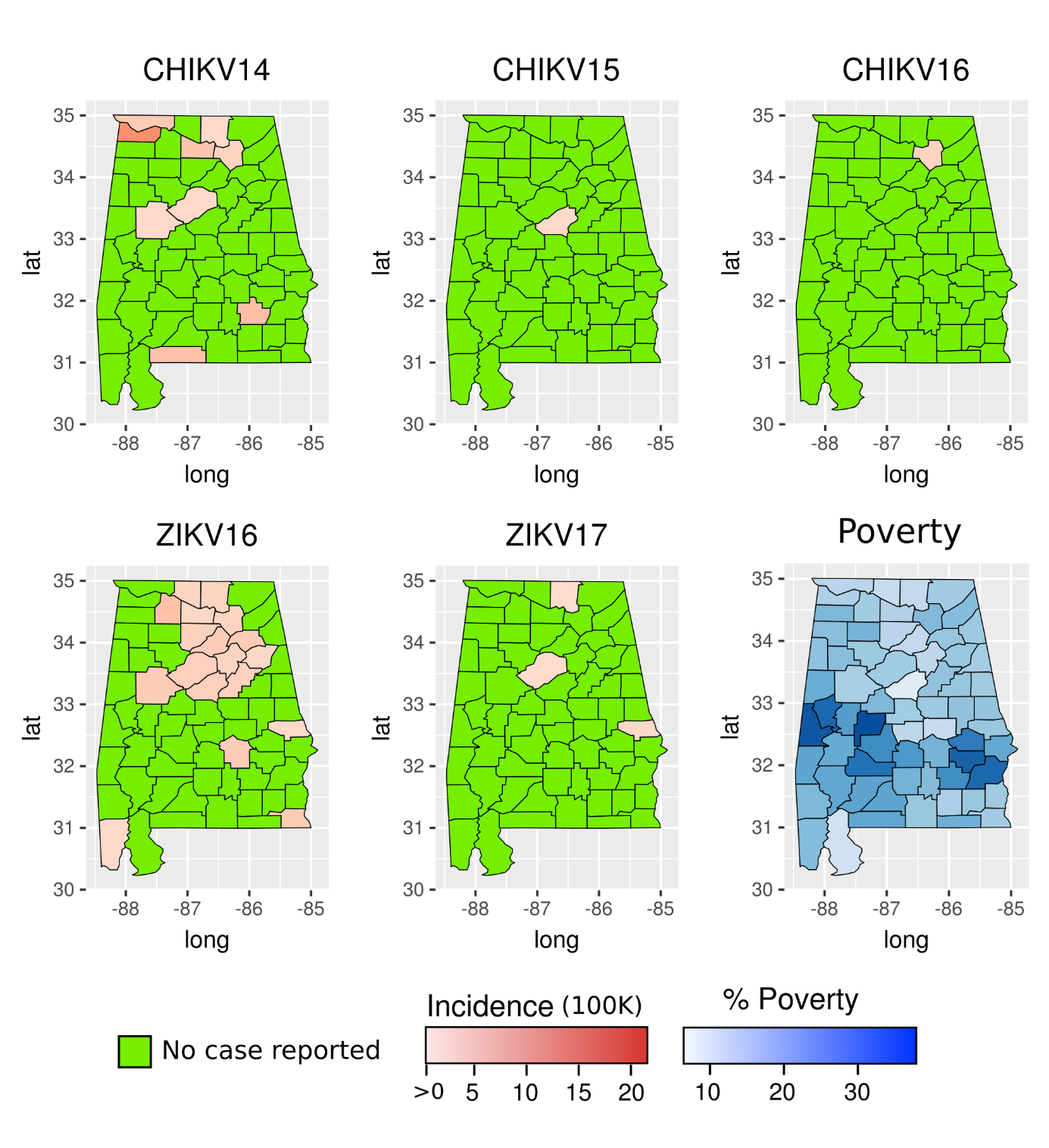


**Fig C. Incidence of zika (ZIKV) and chicungunya (CHIKV) at county level in Alabama from 2007 to 2017.** The maps show incidence of DENV in the years when cases were reported. The figure also includes the percentage of the population living in low socio-economic status at county level based on census data. The maps were created using public available county boundaries downloaded from [www.usgs.gov](http://www.usgs.gov) processed with the R software [1].


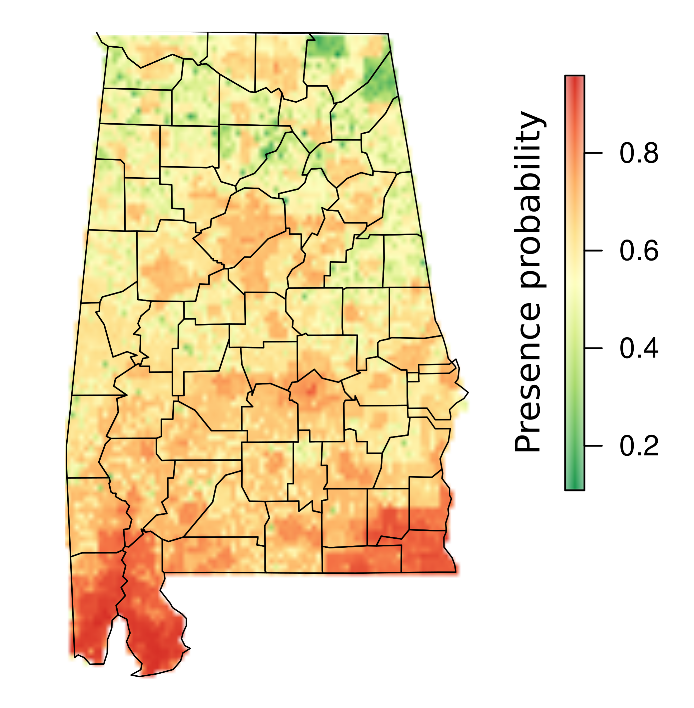


**Fig D. Presence probability of *Ae. aegypti* in 2015.** The map was created using available county boundaries downloaded from [www.usgs.gov](http://www.usgs.gov) and data from the paper written by Kraemer and colleagues [2] (available at: https://malariaatlas.org/) processed with the R software [1].

**References**

1. R Core Team. R: A language and environment for statistical computing. R Foundation for Statistical Computing, Vienna, Austria. 2019. URL <http://www.R-project.org/>.
2. Kraemer MU, Sinka ME, Duda KA, Mylne AQ, Shearer FM, Barker CM. The global distribution of the arbovirus vectors *Aedes aegypti* and *Ae. albopictus*. eLife. 2015; 4:e08347.
